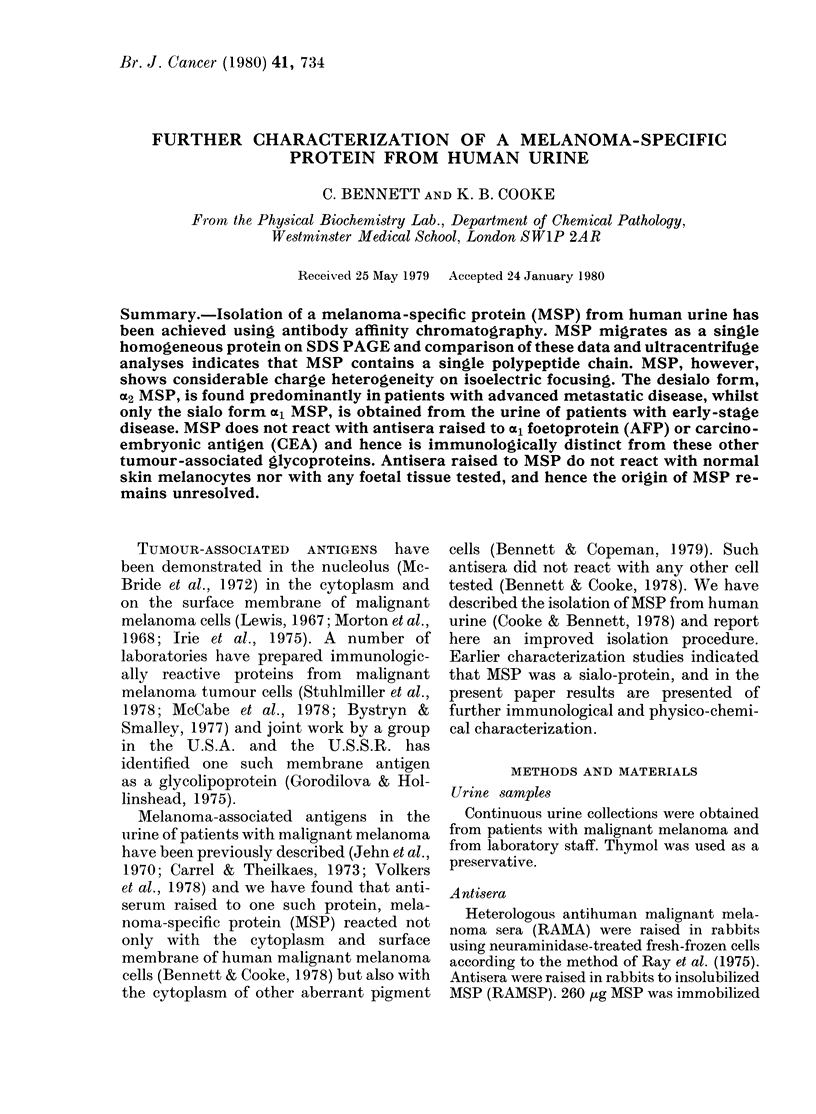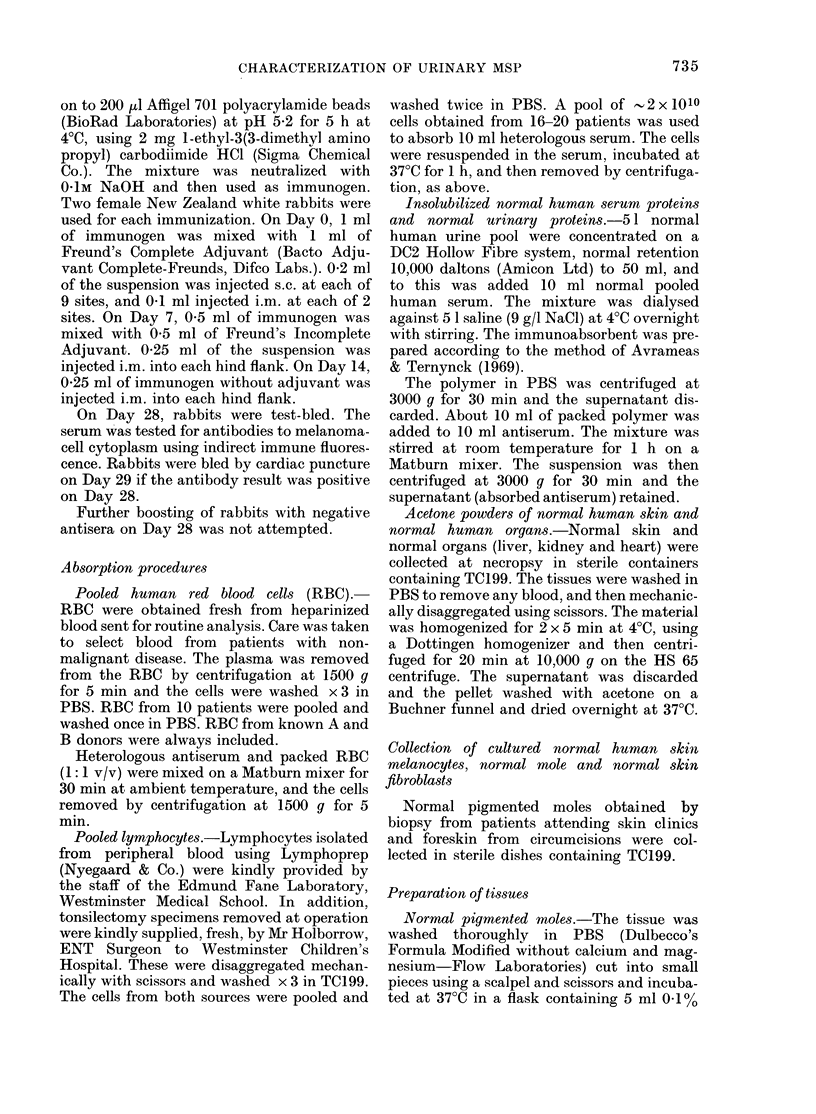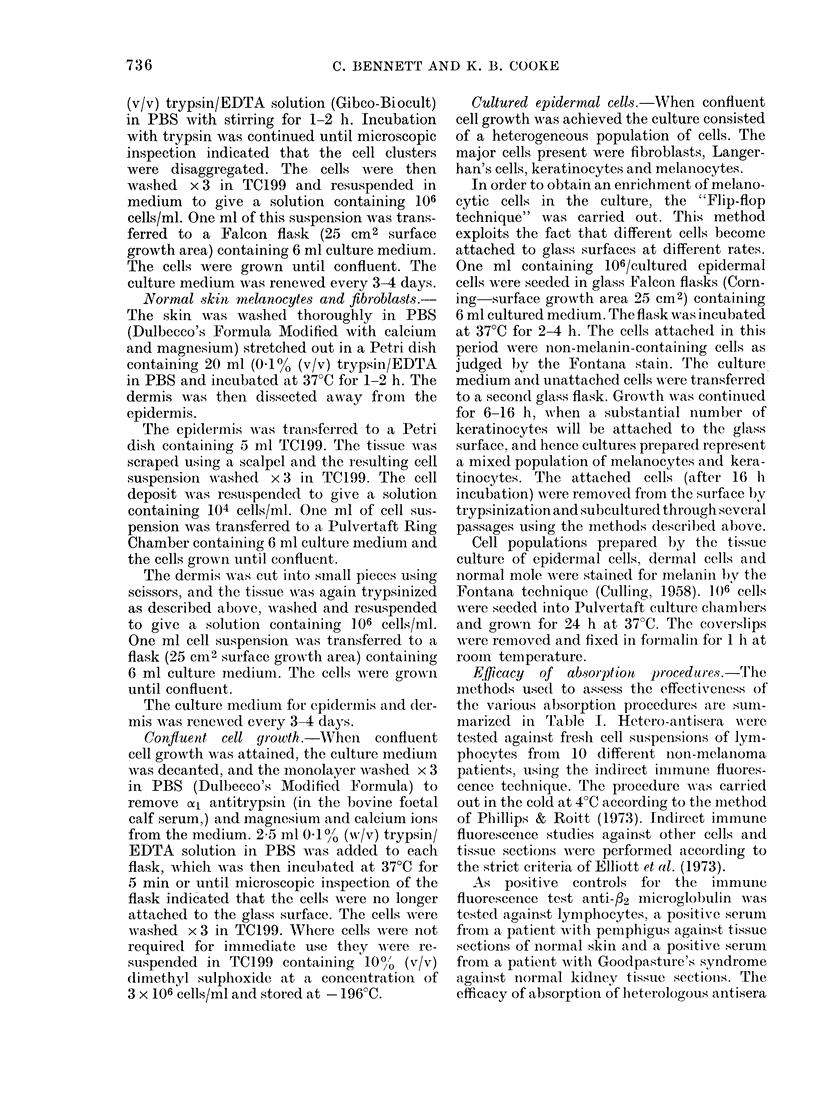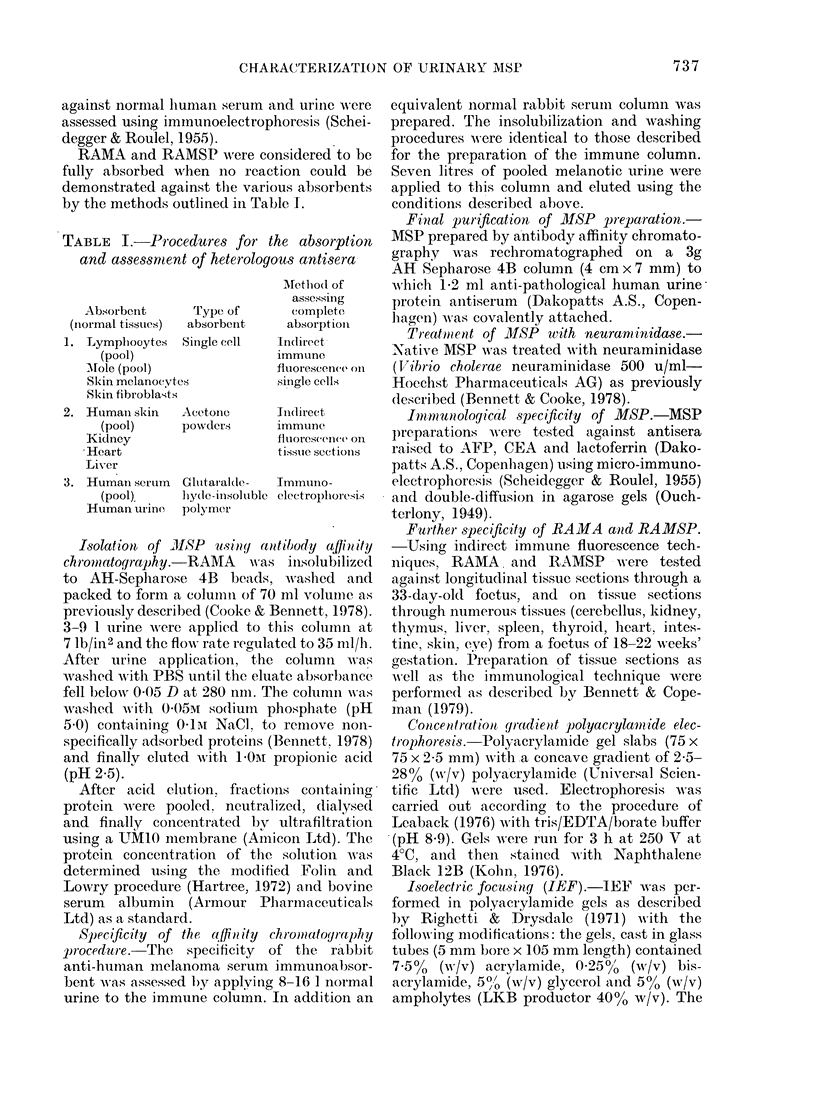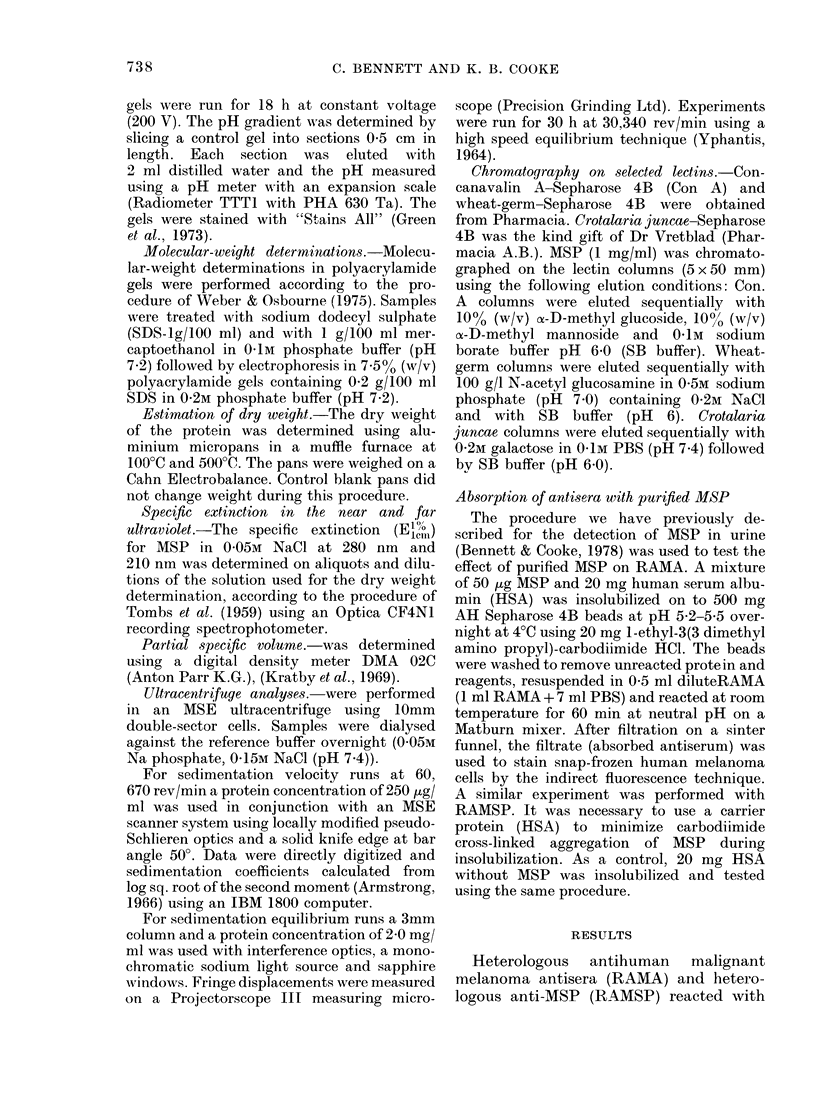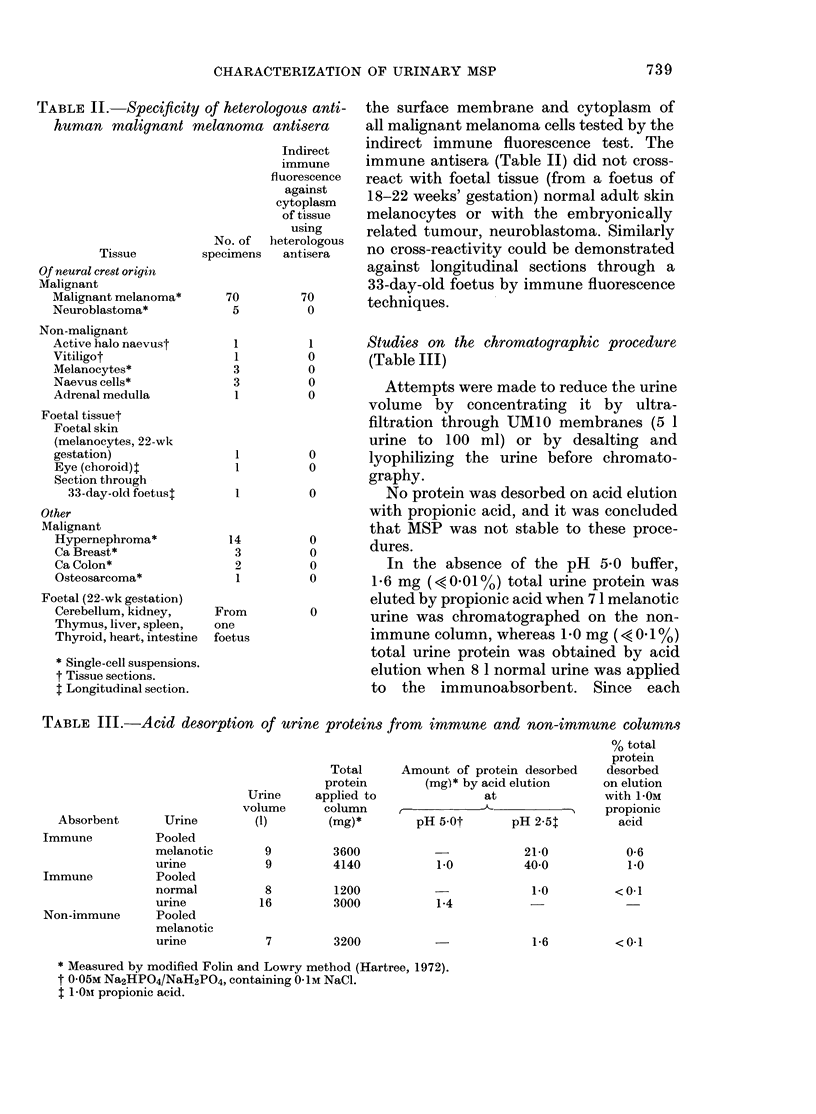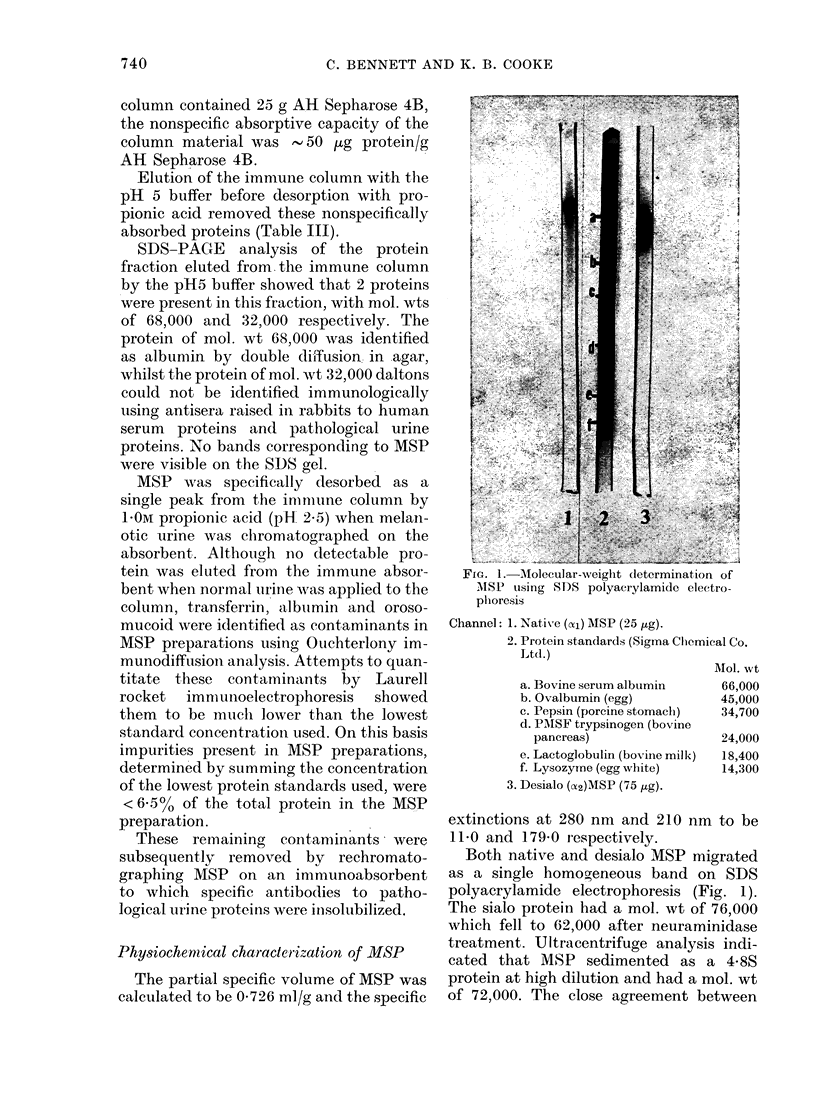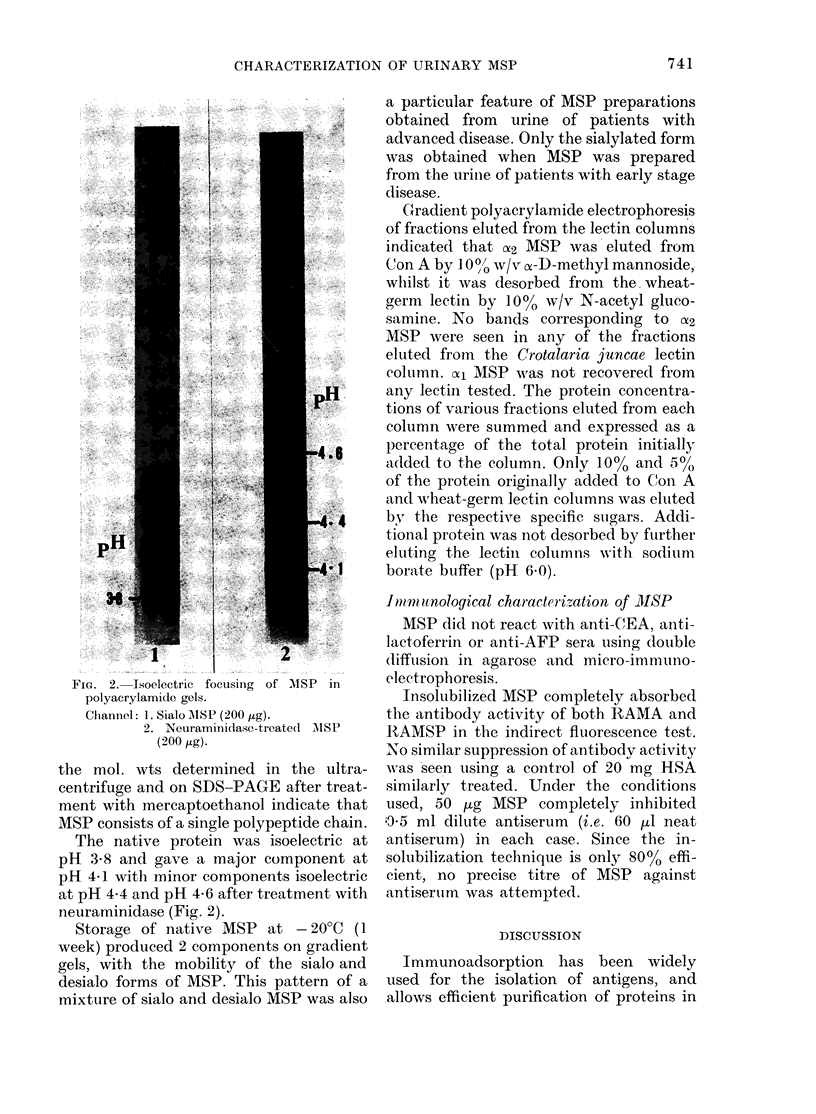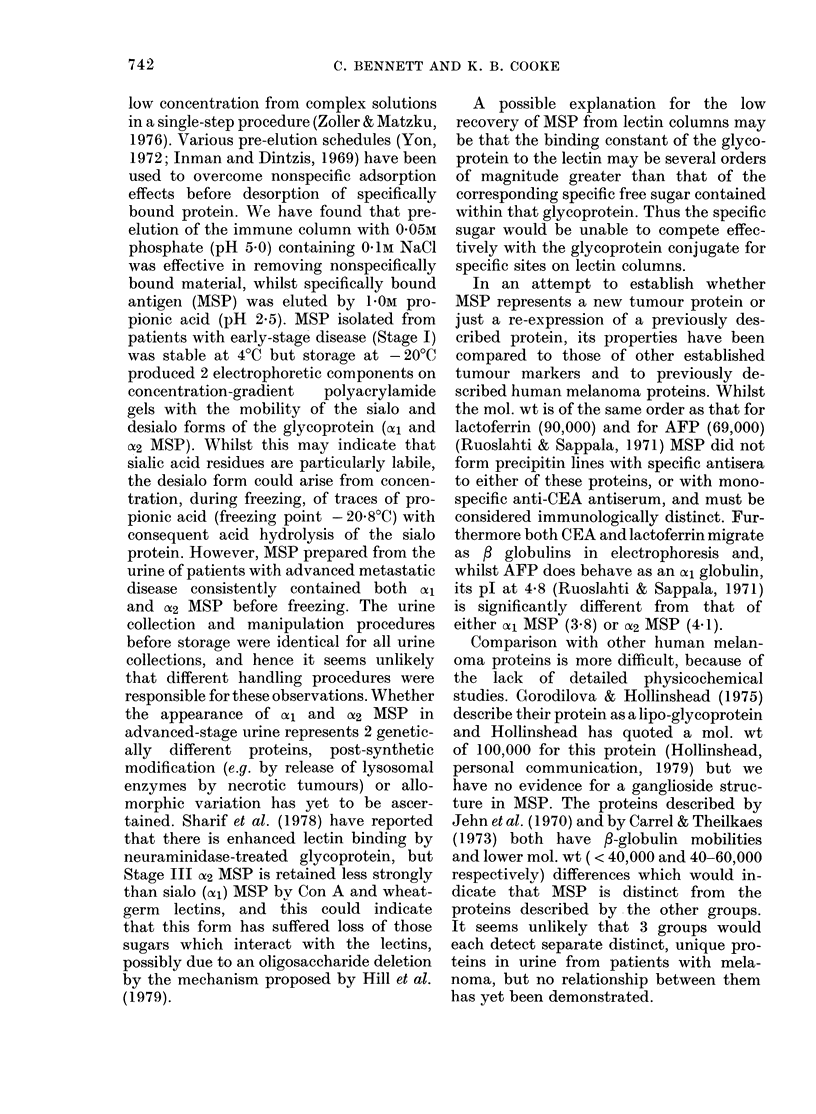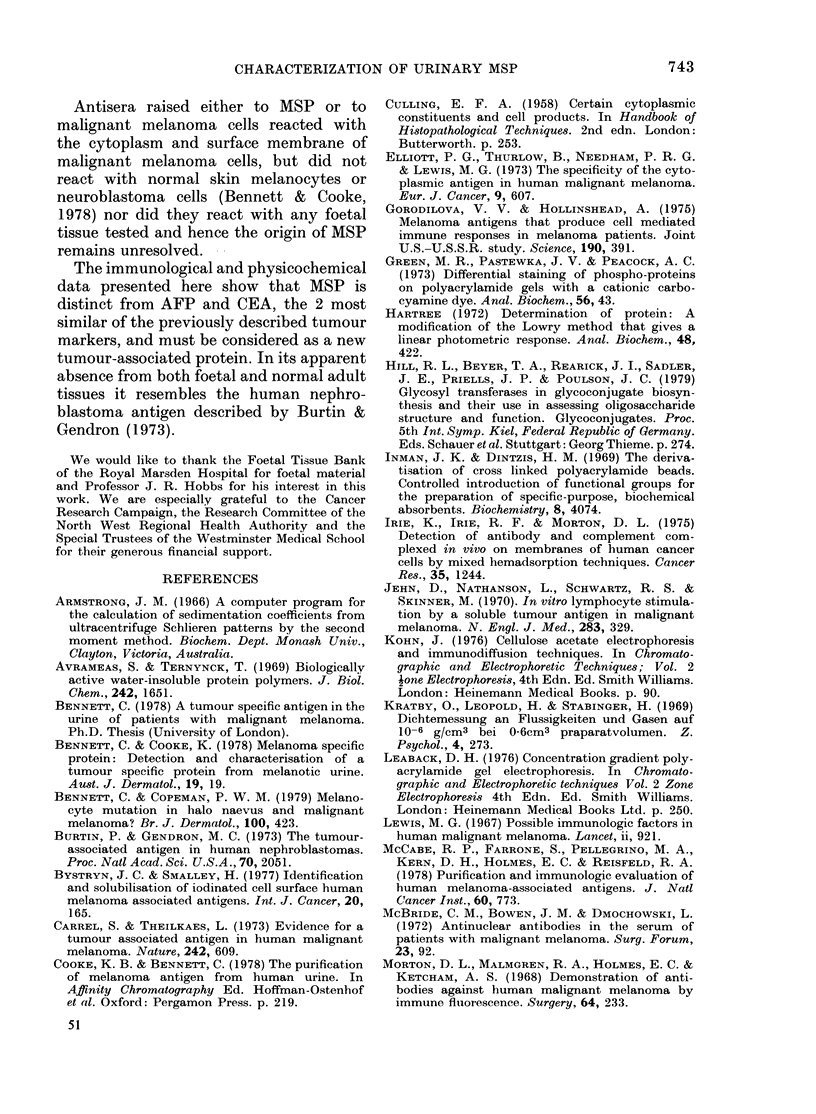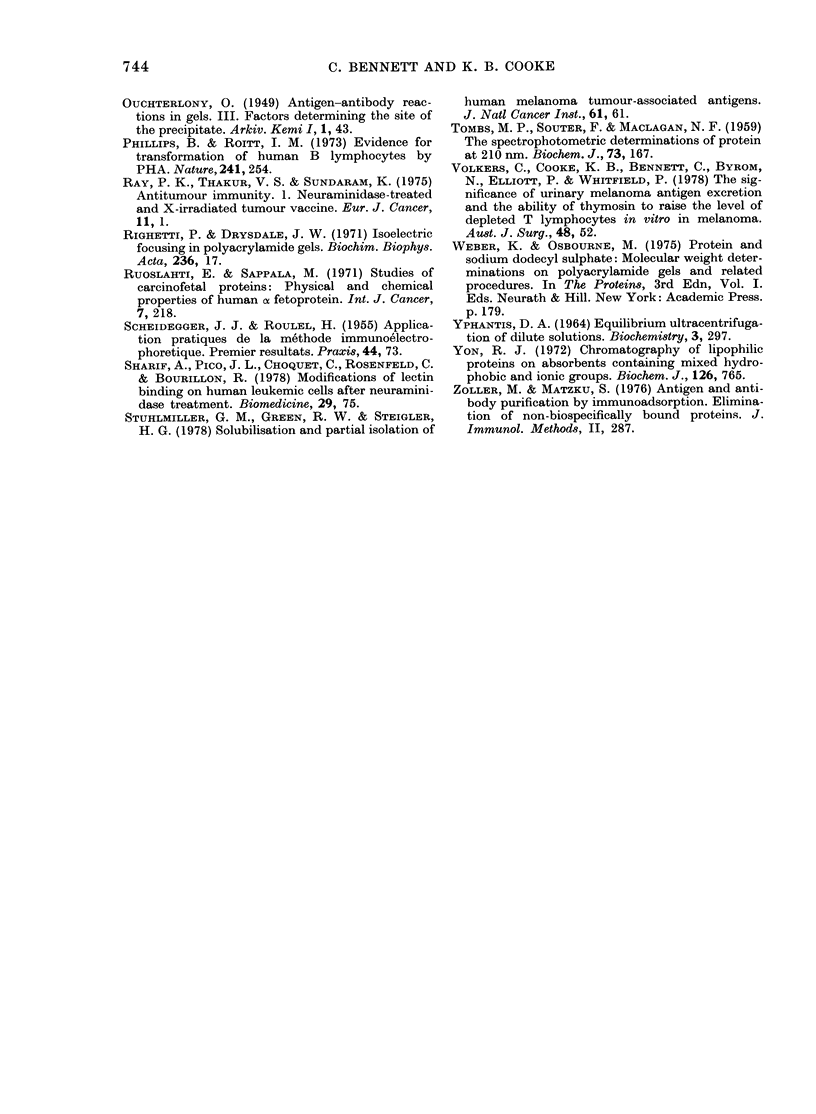# Further characterization of a melanoma-specific protein from human urine.

**DOI:** 10.1038/bjc.1980.135

**Published:** 1980-05

**Authors:** C. Bennett, K. B. Cooke

## Abstract

**Images:**


Br. J. Cancer (1980) 41, 734

### FURTHER CHARACTERIZATION OF A MELANOMA-SPECIFIC PROTEIN FROM HUMAN URINE

C. BENNETT AND K. B. COOKE

*From the Physical Biochemistry Lab., Department of Chemical Pathology, Westminster Medical School, London SW1P 2AR*

Received 25 May 1979 Accepted 24 January 1980

**Summary.—Isolation of a melanoma-specific protein (MSP) from human urine has been achieved using antibody affinity chromatography. MSP migrates as a single homogeneous protein on SDS PAGE and comparison of these data and ultracentrifuge analyses indicates that MSP contains a single polypeptide chain. MSP, however, shows considerable charge heterogeneity on isoelectric focusing. The desialo form, $\alpha_2$ MSP, is found predominantly in patients with advanced metastatic disease, whilst only the sialo form $\alpha_1$ MSP, is obtained from the urine of patients with early-stage disease. MSP does not react with antisera raised to $\alpha_1$ foetoprotein (AFP) or carcino-embryonic antigen (CEA) and hence is immunologically distinct from these other tumour-associated glycoproteins. Antisera raised to MSP do not react with normal skin melanocytes nor with any foetal tissue tested, and hence the origin of MSP remains unresolved.**

TUMOUR-ASSOCIATED ANTIGENS have been demonstrated in the nucleolus (McBride *et al.*, 1972) in the cytoplasm and on the surface membrane of malignant melanoma cells (Lewis, 1967; Morton *et al.*, 1968; Irie *et al.*, 1975). A number of laboratories have prepared immunologically reactive proteins from malignant melanoma tumour cells (Stuhlmiller *et al.*, 1978; McCabe *et al.*, 1978; Bystryn & Smalley, 1977) and joint work by a group in the U.S.A. and the U.S.S.R. has identified one such membrane antigen as a glycolipoprotein (Gorodilova & Hollinshead, 1975).

Melanoma-associated antigens in the urine of patients with malignant melanoma have been previously described (Jehn *et al.*, 1970; Carrel & Theilkaes, 1973; Volkers *et al.*, 1978) and we have found that antiserum raised to one such protein, melanoma-specific protein (MSP) reacted not only with the cytoplasm and surface membrane of human malignant melanoma cells (Bennett & Cooke, 1978) but also with the cytoplasm of other aberrant pigment cells (Bennett & Copeman, 1979). Such antisera did not react with any other cell tested (Bennett & Cooke, 1978). We have described the isolation of MSP from human urine (Cooke & Bennett, 1978) and report here an improved isolation procedure. Earlier characterization studies indicated that MSP was a sialo-protein, and in the present paper results are presented of further immunological and physico-chemical characterization.

### METHODS AND MATERIALS

### *Urine samples*

Continuous urine collections were obtained from patients with malignant melanoma and from laboratory staff. Thymol was used as a preservative.

### *Antisera*

Heterologous antihuman malignant melanoma sera (RAMA) were raised in rabbits using neuraminidase-treated fresh-frozen cells according to the method of Ray *et al.* (1975). Antisera were raised in rabbits to insolubilized MSP (RAMSP). 260 μg MSP was immobilized on to 200 $\mu$l Affigel 701 polyacrylamide beads (BioRad Laboratories) at pH 5·2 for 5 h at 4°C, using 2 mg 1-ethyl-3(3-dimethyl amino propyl) carbodiimide HCl (Sigma Chemical Co.). The mixture was neutralized with 0·1M NaOH and then used as immunogen. Two female New Zealand white rabbits were used for each immunization. On Day 0, 1 ml of immunogen was mixed with 1 ml of Freund's Complete Adjuvant (Bacto Adjuvant Complete-Freunds, Difco Labs.). 0·2 ml of the suspension was injected s.c. at each of 9 sites, and 0·1 ml injected i.m. at each of 2 sites. On Day 7, 0·5 ml of immunogen was mixed with 0·5 ml of Freund's Incomplete Adjuvant. 0·25 ml of the suspension was injected i.m. into each hind flank. On Day 14, 0·25 ml of immunogen without adjuvant was injected i.m. into each hind flank.

On Day 28, rabbits were test-bled. The serum was tested for antibodies to melanoma-cell cytoplasm using indirect immune fluorescence. Rabbits were bled by cardiac puncture on Day 29 if the antibody result was positive on Day 28.

Further boosting of rabbits with negative antisera on Day 28 was not attempted.

*Absorption procedures*

*Pooled human red blood cells* (RBC).—RBC were obtained fresh from heparinized blood sent for routine analysis. Care was taken to select blood from patients with non-malignant disease. The plasma was removed from the RBC by centrifugation at 1500 *g* for 5 min and the cells were washed ×3 in PBS. RBC from 10 patients were pooled and washed once in PBS. RBC from known A and B donors were always included.

Heterologous antiserum and packed RBC (1:1 v/v) were mixed on a Matburn mixer for 30 min at ambient temperature, and the cells removed by centrifugation at 1500 *g* for 5 min.

*Pooled lymphocytes*.—Lymphocytes isolated from peripheral blood using Lymphoprep (Nyegaard & Co.) were kindly provided by the staff of the Edmund Fane Laboratory, Westminster Medical School. In addition, tonsilectomy specimens removed at operation were kindly supplied, fresh, by Mr Holborrow, ENT Surgeon to Westminster Children's Hospital. These were disaggregated mechanically with scissors and washed ×3 in TC199. The cells from both sources were pooled and washed twice in PBS. A pool of $\sim 2 \times 10^{10}$ cells obtained from 16–20 patients was used to absorb 10 ml heterologous serum. The cells were resuspended in the serum, incubated at 37°C for 1 h, and then removed by centrifugation, as above.

*Insolubilized normal human serum proteins and normal urinary proteins*.—5 l normal human urine pool were concentrated on a DC2 Hollow Fibre system, normal retention 10,000 daltons (Amicon Ltd) to 50 ml, and to this was added 10 ml normal pooled human serum. The mixture was dialysed against 5 l saline (9 g/l NaCl) at 4°C overnight with stirring. The immunoabsorbent was prepared according to the method of Avrameas & Ternynck (1969).

The polymer in PBS was centrifuged at 3000 *g* for 30 min and the supernatant discarded. About 10 ml of packed polymer was added to 10 ml antiserum. The mixture was stirred at room temperature for 1 h on a Matburn mixer. The suspension was then centrifuged at 3000 *g* for 30 min and the supernatant (absorbed antiserum) retained.

*Acetone powders of normal human skin and normal human organs*.—Normal skin and normal organs (liver, kidney and heart) were collected at necropsy in sterile containers containing TC199. The tissues were washed in PBS to remove any blood, and then mechanically disaggregated using scissors. The material was homogenized for 2×5 min at 4°C, using a Dottingen homogenizer and then centrifuged for 20 min at 10,000 *g* on the HS 65 centrifuge. The supernatant was discarded and the pellet washed with acetone on a Buchner funnel and dried overnight at 37°C.

*Collection of cultured normal human skin melanocytes, normal mole and normal skin fibroblasts*

Normal pigmented moles obtained by biopsy from patients attending skin clinics and foreskin from circumcisions were collected in sterile dishes containing TC199.

*Preparation of tissues*

*Normal pigmented moles*.—The tissue was washed thoroughly in PBS (Dulbecco's Formula Modified without calcium and magnesium—Flow Laboratories) cut into small pieces using a scalpel and scissors and incubated at 37°C in a flask containing 5 ml 0·1%

(v/v) trypsin/EDTA solution (Gibco-Biocult) in PBS with stirring for 1–2 h. Incubation with trypsin was continued until microscopic inspection indicated that the cell clusters were disaggregated. The cells were then washed ×3 in TC199 and resuspended in medium to give a solution containing $10^6$ cells/ml. One ml of this suspension was transferred to a Falcon flask (25 $cm^2$ surface growth area) containing 6 ml culture medium. The cells were grown until confluent. The culture medium was renewed every 3–4 days.

*Normal skin melanocytes and fibroblasts.*—The skin was washed thoroughly in PBS (Dulbecco's Formula Modified with calcium and magnesium) stretched out in a Petri dish containing 20 ml (0·1% (v/v) trypsin/EDTA in PBS and incubated at 37°C for 1–2 h. The dermis was then dissected away from the epidermis.

The epidermis was transferred to a Petri dish containing 5 ml TC199. The tissue was scraped using a scalpel and the resulting cell suspension washed ×3 in TC199. The cell deposit was resuspended to give a solution containing $10^4$ cells/ml. One ml of cell suspension was transferred to a Pulvertaft Ring Chamber containing 6 ml culture medium and the cells grown until confluent.

The dermis was cut into small pieces using scissors, and the tissue was again trypsinized as described above, washed and resuspended to give a solution containing $10^6$ cells/ml. One ml cell suspension was transferred to a flask (25 $cm^2$ surface growth area) containing 6 ml culture medium. The cells were grown until confluent.

The culture medium for epidermis and dermis was renewed every 3–4 days.

*Confluent cell growth.*—When confluent cell growth was attained, the culture medium was decanted, and the monolayer washed ×3 in PBS (Dulbecco's Modified Formula) to remove $\alpha_1$ antitrypsin (in the bovine foetal calf serum,) and magnesium and calcium ions from the medium. 2·5 ml 0·1% (w/v) trypsin/EDTA solution in PBS was added to each flask, which was then incubated at 37°C for 5 min or until microscopic inspection of the flask indicated that the cells were no longer attached to the glass surface. The cells were washed ×3 in TC199. Where cells were not required for immediate use they were resuspended in TC199 containing 10% (v/v) dimethyl sulphoxide at a concentration of $3 \times 10^6$ cells/ml and stored at −196°C.

*Cultured epidermal cells.*—When confluent cell growth was achieved the culture consisted of a heterogeneous population of cells. The major cells present were fibroblasts, Langerhan's cells, keratinocytes and melanocytes.

In order to obtain an enrichment of melanocytic cells in the culture, the "Flip-flop technique" was carried out. This method exploits the fact that different cells become attached to glass surfaces at different rates. One ml containing $10^6$/cultured epidermal cells were seeded in glass Falcon flasks (Corning—surface growth area 25 $cm^2$) containing 6 ml cultured medium. The flask was incubated at 37°C for 2–4 h. The cells attached in this period were non-melanin-containing cells as judged by the Fontana stain. The culture medium and unattached cells were transferred to a second glass flask. Growth was continued for 6–16 h, when a substantial number of keratinocytes will be attached to the glass surface, and hence cultures prepared represent a mixed population of melanocytes and keratinocytes. The attached cells (after 16 h incubation) were removed from the surface by trypsinization and subcultured through several passages using the methods described above.

Cell populations prepared by the tissue culture of epidermal cells, dermal cells and normal mole were stained for melanin by the Fontana technique (Culling, 1958). $10^6$ cells were seeded into Pulvertaft culture chambers and grown for 24 h at 37°C. The coverslips were removed and fixed in formalin for 1 h at room temperature.

*Efficacy of absorption procedures.*—The methods used to assess the effectiveness of the various absorption procedures are summarized in Table I. Hetero-antisera were tested against fresh cell suspensions of lymphocytes from 10 different non-melanoma patients, using the indirect immune fluorescence technique. The procedure was carried out in the cold at 4°C according to the method of Phillips & Roitt (1973). Indirect immune fluorescence studies against other cells and tissue sections were performed according to the strict criteria of Elliott *et al.* (1973).

As positive controls for the immune fluorescence test anti-$\beta_2$ microglobulin was tested against lymphocytes, a positive serum from a patient with pemphigus against tissue sections of normal skin and a positive serum from a patient with Goodpasture's syndrome against normal kidney tissue sections. The efficacy of absorption of heterologous antisera against normal human serum and urine were assessed using immunoelectrophoresis (Scheidegger & Roulel, 1955).

RAMA and RAMSP were considered to be fully absorbed when no reaction could be demonstrated against the various absorbents by the methods outlined in Table I.

TABLE I.—*Procedures for the absorption and assessment of heterologous antisera*

| Absorbent (normal tissues) | Type of absorbent | Method of assessing complete absorption |
|---|---|---|
| 1. Lymphocytes (pool)<br>Mole (pool)<br>Skin melanocytes<br>Skin fibroblasts | Single cell | Indirect immune fluorescence on single cells |
| 2. Human skin (pool)<br>Kidney<br>Heart<br>Liver | Acetone powders | Indirect immune fluorescence on tissue sections |
| 3. Human serum (pool)<br>Human urine | Glutaraldehyde-insoluble polymer | Immuno-electrophoresis |

*Isolation of MSP using antibody affinity chromatography.*—RAMA was insolubilized to AH-Sepharose 4B beads, washed and packed to form a column of 70 ml volume as previously described (Cooke & Bennett, 1978). 3–9 l urine were applied to this column at 7 lb/in$^2$ and the flow rate regulated to 35 ml/h. After urine application, the column was washed with PBS until the eluate absorbance fell below 0·05 $D$ at 280 nm. The column was washed with 0·05M sodium phosphate (pH 5·0) containing 0·1M NaCl, to remove non-specifically adsorbed proteins (Bennett, 1978) and finally eluted with 1·0M propionic acid (pH 2·5).

After acid elution, fractions containing protein were pooled, neutralized, dialysed and finally concentrated by ultrafiltration using a UM10 membrane (Amicon Ltd). The protein concentration of the solution was determined using the modified Folin and Lowry procedure (Hartree, 1972) and bovine serum albumin (Armour Pharmaceuticals Ltd) as a standard.

*Specificity of the affinity chromatography procedure.*—The specificity of the rabbit anti-human melanoma serum immunoabsorbent was assessed by applying 8–16 l normal urine to the immune column. In addition an equivalent normal rabbit serum column was prepared. The insolubilization and washing procedures were identical to those described for the preparation of the immune column. Seven litres of pooled melanotic urine were applied to this column and eluted using the conditions described above.

*Final purification of MSP preparation.*—MSP prepared by antibody affinity chromatography was rechromatographed on a 3g AH Sepharose 4B column (4 cm × 7 mm) to which 1·2 ml anti-pathological human urine protein antiserum (Dakopatts A.S., Copenhagen) was covalently attached.

*Treatment of MSP with neuraminidase.*—Native MSP was treated with neuraminidase (*Vibrio cholerae* neuraminidase 500 u/ml—Hoechst Pharmaceuticals AG) as previously described (Bennett & Cooke, 1978).

*Immunological specificity of MSP.*—MSP preparations were tested against antisera raised to AFP, CEA and lactoferrin (Dakopatts A.S., Copenhagen) using micro-immunoelectrophoresis (Scheidegger & Roulel, 1955) and double-diffusion in agarose gels (Ouchterlony, 1949).

*Further specificity of RAMA and RAMSP.*—Using indirect immune fluorescence techniques, RAMA and RAMSP were tested against longitudinal tissue sections through a 33-day-old foetus, and on tissue sections through numerous tissues (cerebellus, kidney, thymus, liver, spleen, thyroid, heart, intestine, skin, eye) from a foetus of 18–22 weeks' gestation. Preparation of tissue sections as well as the immunological technique were performed as described by Bennett & Copeman (1979).

*Concentration gradient polyacrylamide electrophoresis.*—Polyacrylamide gel slabs (75 × 75 × 2·5 mm) with a concave gradient of 2·5–28% (w/v) polyacrylamide (Universal Scientific Ltd) were used. Electrophoresis was carried out according to the procedure of Leaback (1976) with tris/EDTA/borate buffer (pH 8·9). Gels were run for 3 h at 250 V at 4°C, and then stained with Naphthalene Black 12B (Kohn, 1976).

*Isoelectric focusing (IEF).*—IEF was performed in polyacrylamide gels as described by Righetti & Drysdale (1971) with the following modifications: the gels, cast in glass tubes (5 mm bore × 105 mm length) contained 7·5% (w/v) acrylamide, 0·25% (w/v) bis-acrylamide, 5% (w/v) glycerol and 5% (w/v) ampholytes (LKB productor 40% w/v). The gels were run for 18 h at constant voltage (200 V). The pH gradient was determined by slicing a control gel into sections 0·5 cm in length. Each section was eluted with 2 ml distilled water and the pH measured using a pH meter with an expansion scale (Radiometer TTT1 with PHA 630 Ta). The gels were stained with "Stains All" (Green *et al.*, 1973).

*Molecular-weight determinations.*—Molecular-weight determinations in polyacrylamide gels were performed according to the procedure of Weber & Osbourne (1975). Samples were treated with sodium dodecyl sulphate (SDS-1g/100 ml) and with 1 g/100 ml mercaptoethanol in 0·1M phosphate buffer (pH 7·2) followed by electrophoresis in 7·5% (w/v) polyacrylamide gels containing 0·2 g/100 ml SDS in 0·2M phosphate buffer (pH 7·2).

*Estimation of dry weight.*—The dry weight of the protein was determined using aluminium micropans in a muffle furnace at 100°C and 500°C. The pans were weighed on a Cahn Electrobalance. Control blank pans did not change weight during this procedure.

*Specific extinction in the near and far ultraviolet.*—The specific extinction ($E^{1\%}_{1cm}$) for MSP in 0·05M NaCl at 280 nm and 210 nm was determined on aliquots and dilutions of the solution used for the dry weight determination, according to the procedure of Tombs *et al.* (1959) using an Optica CF4N1 recording spectrophotometer.

*Partial specific volume.*—was determined using a digital density meter DMA 02C (Anton Parr K.G.), (Kratby *et al.*, 1969).

*Ultracentrifuge analyses.*—were performed in an MSE ultracentrifuge using 10mm double-sector cells. Samples were dialysed against the reference buffer overnight (0·05M Na phosphate, 0·15M NaCl (pH 7·4)).

For sedimentation velocity runs at 60, 670 rev/min a protein concentration of 250 μg/ml was used in conjunction with an MSE scanner system using locally modified pseudo-Schlieren optics and a solid knife edge at bar angle 50°. Data were directly digitized and sedimentation coefficients calculated from log sq. root of the second moment (Armstrong, 1966) using an IBM 1800 computer.

For sedimentation equilibrium runs a 3mm column and a protein concentration of 2·0 mg/ml was used with interference optics, a monochromatic sodium light source and sapphire windows. Fringe displacements were measured on a Projectorscope III measuring microscope (Precision Grinding Ltd). Experiments were run for 30 h at 30,340 rev/min using a high speed equilibrium technique (Yphantis, 1964).

*Chromatography on selected lectins.*—Concanavalin A–Sepharose 4B (Con A) and wheat-germ–Sepharose 4B were obtained from Pharmacia. *Crotalaria juncae*–Sepharose 4B was the kind gift of Dr Vretblad (Pharmacia A.B.). MSP (1 mg/ml) was chromatographed on the lectin columns (5 × 50 mm) using the following elution conditions: Con. A columns were eluted sequentially with 10% (w/v) α-D-methyl glucoside, 10% (w/v) α-D-methyl mannoside and 0·1M sodium borate buffer pH 6·0 (SB buffer). Wheat-germ columns were eluted sequentially with 100 g/l N-acetyl glucosamine in 0·5M sodium phosphate (pH 7·0) containing 0·2M NaCl and with SB buffer (pH 6). *Crotalaria juncae* columns were eluted sequentially with 0·2M galactose in 0·1M PBS (pH 7·4) followed by SB buffer (pH 6·0).

### *Absorption of antisera with purified MSP*

The procedure we have previously described for the detection of MSP in urine (Bennett & Cooke, 1978) was used to test the effect of purified MSP on RAMA. A mixture of 50 μg MSP and 20 mg human serum albumin (HSA) was insolubilized on to 500 mg AH Sepharose 4B beads at pH 5·2–5·5 overnight at 4°C using 20 mg 1-ethyl-3(3 dimethyl amino propyl)-carbodiimide HCl. The beads were washed to remove unreacted protein and reagents, resuspended in 0·5 ml diluteRAMA (1 ml RAMA + 7 ml PBS) and reacted at room temperature for 60 min at neutral pH on a Matburn mixer. After filtration on a sinter funnel, the filtrate (absorbed antiserum) was used to stain snap-frozen human melanoma cells by the indirect fluorescence technique. A similar experiment was performed with RAMSP. It was necessary to use a carrier protein (HSA) to minimize carbodiimide cross-linked aggregation of MSP during insolubilization. As a control, 20 mg HSA without MSP was insolubilized and tested using the same procedure.

### RESULTS

Heterologous antihuman malignant melanoma antisera (RAMA) and heterologous anti-MSP (RAMSP) reacted with

TABLE II.—*Specificity of heterologous anti-human malignant melanoma antisera*

| Tissue | No. of specimens | Indirect immune fluorescence against cytoplasm of tissue using heterologous antisera |
|---|---|---|
| *Of neural crest origin* | | |
| Malignant | | |
| Malignant melanoma* | 70 | 70 |
| Neuroblastoma* | 5 | 0 |
| Non-malignant | | |
| Active halo naevus† | 1 | 1 |
| Vitiligo† | 1 | 0 |
| Melanocytes* | 3 | 0 |
| Naevus cells* | 3 | 0 |
| Adrenal medulla | 1 | 0 |
| Foetal tissue† | | |
| Foetal skin (melanocytes, 22-wk gestation) | 1 | 0 |
| Eye (choroid)‡ | 1 | 0 |
| Section through 33-day-old foetus‡ | 1 | 0 |
| *Other* | | |
| Malignant | | |
| Hypernephroma* | 14 | 0 |
| Ca Breast* | 3 | 0 |
| Ca Colon* | 2 | 0 |
| Osteosarcoma* | 1 | 0 |
| Foetal (22-wk gestation) | | |
| Cerebellum, kidney, Thymus, liver, spleen, Thyroid, heart, intestine | From one foetus | 0 |

* Single-cell suspensions.

† Tissue sections.

‡ Longitudinal section.

the surface membrane and cytoplasm of all malignant melanoma cells tested by the indirect immune fluorescence test. The immune antisera (Table II) did not cross-react with foetal tissue (from a foetus of 18–22 weeks' gestation) normal adult skin melanocytes or with the embryonically related tumour, neuroblastoma. Similarly no cross-reactivity could be demonstrated against longitudinal sections through a 33-day-old foetus by immune fluorescence techniques.

*Studies on the chromatographic procedure* (Table III)

Attempts were made to reduce the urine volume by concentrating it by ultra-filtration through UM10 membranes (5 l urine to 100 ml) or by desalting and lyophilizing the urine before chromatography.

No protein was desorbed on acid elution with propionic acid, and it was concluded that MSP was not stable to these procedures.

In the absence of the pH 5·0 buffer, 1·6 mg ($\ll$0·01%) total urine protein was eluted by propionic acid when 7 l melanotic urine was chromatographed on the non-immune column, whereas 1·0 mg ($\ll$0·1%) total urine protein was obtained by acid elution when 8 l normal urine was applied to the immunoabsorbent. Since each

TABLE III.—*Acid desorption of urine proteins from immune and non-immune columns*

| Absorbent | Urine | Urine volume (l) | Total protein applied to column (mg)* | Amount of protein desorbed (mg)* by acid elution at | | % total protein desorbed on elution with 1·0M propionic acid |
|---|---|---|---|---|---|---|
| | | | | pH 5·0† | pH 2·5‡ | |
| Immune | Pooled melanotic urine | 9 | 3600 | — | 21·0 | 0·6 |
| | | 9 | 4140 | 1·0 | 40·0 | 1·0 |
| Immune | Pooled normal urine | 8 | 1200 | — | 1·0 | <0·1 |
| | | 16 | 3000 | 1·4 | — | — |
| Non-immune | Pooled melanotic urine | 7 | 3200 | — | 1·6 | <0·1 |

* Measured by modified Folin and Lowry method (Hartree, 1972).
† 0·05M $Na_2HPO_4/NaH_2PO_4$, containing 0·1M NaCl.
‡ 1·0M propionic acid.

column contained 25 g AH Sepharose 4B, the nonspecific absorptive capacity of the column material was ~50 μg protein/g AH Sepharose 4B.

Elution of the immune column with the pH 5 buffer before desorption with propionic acid removed these nonspecifically absorbed proteins (Table III).

SDS-PAGE analysis of the protein fraction eluted from the immune column by the pH5 buffer showed that 2 proteins were present in this fraction, with mol. wts of 68,000 and 32,000 respectively. The protein of mol. wt 68,000 was identified as albumin by double diffusion in agar, whilst the protein of mol. wt 32,000 daltons could not be identified immunologically using antisera raised in rabbits to human serum proteins and pathological urine proteins. No bands corresponding to MSP were visible on the SDS gel.

MSP was specifically desorbed as a single peak from the immune column by 1·0M propionic acid (pH 2·5) when melanotic urine was chromatographed on the absorbent. Although no detectable protein was eluted from the immune absorbent when normal urine was applied to the column, transferrin, albumin and orosomucoid were identified as contaminants in MSP preparations using Ouchterlony immunodiffusion analysis. Attempts to quantitate these contaminants by Laurell rocket immunoelectrophoresis showed them to be much lower than the lowest standard concentration used. On this basis impurities present in MSP preparations, determined by summing the concentration of the lowest protein standards used, were < 6·5% of the total protein in the MSP preparation.

These remaining contaminants were subsequently removed by rechromatographing MSP on an immunoabsorbent to which specific antibodies to pathological urine proteins were insolubilized.

*Physiochemical characterization of MSP*

The partial specific volume of MSP was calculated to be 0·726 ml/g and the specific

FIG. 1.—Molecular-weight determination of MSP using SDS polyacrylamide electrophoresis

Channel: 1. Native ($\alpha_1$) MSP (25 μg).

2. Protein standards (Sigma Chemical Co. Ltd.)

| | Mol. wt |
|---|---|
| a. Bovine serum albumin | 66,000 |
| b. Ovalbumin (egg) | 45,000 |
| c. Pepsin (porcine stomach) | 34,700 |
| d. PMSF trypsinogen (bovine pancreas) | 24,000 |
| e. Lactoglobulin (bovine milk) | 18,400 |
| f. Lysozyme (egg white) | 14,300 |

3. Desialo ($\alpha_2$)MSP (75 μg).

extinctions at 280 nm and 210 nm to be 11·0 and 179·0 respectively.

Both native and desialo MSP migrated as a single homogeneous band on SDS polyacrylamide electrophoresis (Fig. 1). The sialo protein had a mol. wt of 76,000 which fell to 62,000 after neuraminidase treatment. Ultracentrifuge analysis indicated that MSP sedimented as a 4·8S protein at high dilution and had a mol. wt of 72,000. The close agreement between

FIG. 2.—Isoelectric focusing of MSP in polyacrylamide gels.

Channel: 1. Sialo MSP (200 $\mu$g).

2. Neuraminidase-treated MSP (200 $\mu$g).

the mol. wts determined in the ultracentrifuge and on SDS–PAGE after treatment with mercaptoethanol indicate that MSP consists of a single polypeptide chain.

The native protein was isoelectric at pH 3·8 and gave a major component at pH 4·1 with minor components isoelectric at pH 4·4 and pH 4·6 after treatment with neuraminidase (Fig. 2).

Storage of native MSP at $-20°C$ (1 week) produced 2 components on gradient gels, with the mobility of the sialo and desialo forms of MSP. This pattern of a mixture of sialo and desialo MSP was also a particular feature of MSP preparations obtained from urine of patients with advanced disease. Only the sialylated form was obtained when MSP was prepared from the urine of patients with early stage disease.

Gradient polyacrylamide electrophoresis of fractions eluted from the lectin columns indicated that $\alpha_2$ MSP was eluted from Con A by 10% w/v $\alpha$-D-methyl mannoside, whilst it was desorbed from the wheat-germ lectin by 10% w/v N-acetyl glucosamine. No bands corresponding to $\alpha_2$ MSP were seen in any of the fractions eluted from the *Crotalaria juncae* lectin column. $\alpha_1$ MSP was not recovered from any lectin tested. The protein concentrations of various fractions eluted from each column were summed and expressed as a percentage of the total protein initially added to the column. Only 10% and 5% of the protein originally added to Con A and wheat-germ lectin columns was eluted by the respective specific sugars. Additional protein was not desorbed by further eluting the lectin columns with sodium borate buffer (pH 6·0).

### *Immunological characterization of MSP*

MSP did not react with anti-CEA, anti-lactoferrin or anti-AFP sera using double diffusion in agarose and micro-immunoelectrophoresis.

Insolubilized MSP completely absorbed the antibody activity of both RAMA and RAMSP in the indirect fluorescence test. No similar suppression of antibody activity was seen using a control of 20 mg HSA similarly treated. Under the conditions used, 50 $\mu$g MSP completely inhibited 0·5 ml dilute antiserum (*i.e.* 60 $\mu$l neat antiserum) in each case. Since the insolubilization technique is only 80% efficient, no precise titre of MSP against antiserum was attempted.

### DISCUSSION

Immunoadsorption has been widely used for the isolation of antigens, and allows efficient purification of proteins in low concentration from complex solutions in a single-step procedure (Zoller & Matzku, 1976). Various pre-elution schedules (Yon, 1972; Inman and Dintzis, 1969) have been used to overcome nonspecific adsorption effects before desorption of specifically bound protein. We have found that pre-elution of the immune column with 0·05M phosphate (pH 5·0) containing 0·1M NaCl was effective in removing nonspecifically bound material, whilst specifically bound antigen (MSP) was eluted by 1·0M propionic acid (pH 2·5). MSP isolated from patients with early-stage disease (Stage I) was stable at 4°C but storage at $-20$°C produced 2 electrophoretic components on concentration-gradient polyacrylamide gels with the mobility of the sialo and desialo forms of the glycoprotein ($\alpha_1$ and $\alpha_2$ MSP). Whilst this may indicate that sialic acid residues are particularly labile, the desialo form could arise from concentration, during freezing, of traces of propionic acid (freezing point $-20·8$°C) with consequent acid hydrolysis of the sialo protein. However, MSP prepared from the urine of patients with advanced metastatic disease consistently contained both $\alpha_1$ and $\alpha_2$ MSP before freezing. The urine collection and manipulation procedures before storage were identical for all urine collections, and hence it seems unlikely that different handling procedures were responsible for these observations. Whether the appearance of $\alpha_1$ and $\alpha_2$ MSP in advanced-stage urine represents 2 genetically different proteins, post-synthetic modification (*e.g.* by release of lysosomal enzymes by necrotic tumours) or allomorphic variation has yet to be ascertained. Sharif *et al.* (1978) have reported that there is enhanced lectin binding by neuraminidase-treated glycoprotein, but Stage III $\alpha_2$ MSP is retained less strongly than sialo ($\alpha_1$) MSP by Con A and wheat-germ lectins, and this could indicate that this form has suffered loss of those sugars which interact with the lectins, possibly due to an oligosaccharide deletion by the mechanism proposed by Hill *et al.* (1979).

A possible explanation for the low recovery of MSP from lectin columns may be that the binding constant of the glycoprotein to the lectin may be several orders of magnitude greater than that of the corresponding specific free sugar contained within that glycoprotein. Thus the specific sugar would be unable to compete effectively with the glycoprotein conjugate for specific sites on lectin columns.

In an attempt to establish whether MSP represents a new tumour protein or just a re-expression of a previously described protein, its properties have been compared to those of other established tumour markers and to previously described human melanoma proteins. Whilst the mol. wt is of the same order as that for lactoferrin (90,000) and for AFP (69,000) (Ruoslahti & Sappala, 1971) MSP did not form precipitin lines with specific antisera to either of these proteins, or with monospecific anti-CEA antiserum, and must be considered immunologically distinct. Furthermore both CEA and lactoferrin migrate as $\beta$ globulins in electrophoresis and, whilst AFP does behave as an $\alpha_1$ globulin, its pI at 4·8 (Ruoslahti & Sappala, 1971) is significantly different from that of either $\alpha_1$ MSP (3·8) or $\alpha_2$ MSP (4·1).

Comparison with other human melanoma proteins is more difficult, because of the lack of detailed physicochemical studies. Gorodilova & Hollinshead (1975) describe their protein as a lipo-glycoprotein and Hollinshead has quoted a mol. wt of 100,000 for this protein (Hollinshead, personal communication, 1979) but we have no evidence for a ganglioside structure in MSP. The proteins described by Jehn *et al.* (1970) and by Carrel & Theilkaes (1973) both have $\beta$-globulin mobilities and lower mol. wt ($<40,000$ and 40–60,000 respectively) differences which would indicate that MSP is distinct from the proteins described by the other groups. It seems unlikely that 3 groups would each detect separate distinct, unique proteins in urine from patients with melanoma, but no relationship between them has yet been demonstrated.

Antisera raised either to MSP or to malignant melanoma cells reacted with the cytoplasm and surface membrane of malignant melanoma cells, but did not react with normal skin melanocytes or neuroblastoma cells (Bennett & Cooke, 1978) nor did they react with any foetal tissue tested and hence the origin of MSP remains unresolved.

The immunological and physicochemical data presented here show that MSP is distinct from AFP and CEA, the 2 most similar of the previously described tumour markers, and must be considered as a new tumour-associated protein. In its apparent absence from both foetal and normal adult tissues it resembles the human nephroblastoma antigen described by Burtin & Gendron (1973).

We would like to thank the Foetal Tissue Bank of the Royal Marsden Hospital for foetal material and Professor J. R. Hobbs for his interest in this work. We are especially grateful to the Cancer Research Campaign, the Research Committee of the North West Regional Health Authority and the Special Trustees of the Westminster Medical School for their generous financial support.

### REFERENCES

ARMSTRONG, J. M. (1966) A computer program for the calculation of sedimentation coefficients from ultracentrifuge Schlieren patterns by the second moment method. *Biochem. Dept. Monash Univ., Clayton, Victoria, Australia.*

AVRAMEAS, S. & TERNYNCK, T. (1969) Biologically active water-insoluble protein polymers. *J. Biol. Chem.*, **242**, 1651.

BENNETT, C. (1978) A tumour specific antigen in the urine of patients with malignant melanoma. Ph.D. Thesis (University of London).

BENNETT, C. & COOKE, K. (1978) Melanoma specific protein: Detection and characterisation of a tumour specific protein from melanotic urine. *Aust. J. Dermatol.*, **19**, 19.

BENNETT, C. & COPEMAN, P. W. M. (1979) Melanocyte mutation in halo naevus and malignant melanoma? *Br. J. Dermatol.*, **100**, 423.

BURTIN, P. & GENDRON, M. C. (1973) The tumour-associated antigen in human nephroblastomas. *Proc. Natl Acad. Sci. U.S.A.*, **70**, 2051.

BYSTRYN, J. C. & SMALLEY, H. (1977) Identification and solubilisation of iodinated cell surface human melanoma associated antigens. *Int. J. Cancer*, **20**, 165.

CARREL, S. & THEILKAES, L. (1973) Evidence for a tumour associated antigen in human malignant melanoma. *Nature*, **242**, 609.

COOKE, K. B. & BENNETT, C. (1978) The purification of melanoma antigen from human urine. In *Affinity Chromatography* Ed. Hoffman-Ostenhof *et al.* Oxford: Pergamon Press. p. 219.

CULLING, E. F. A. (1958) Certain cytoplasmic constituents and cell products. In *Handbook of Histopathological Techniques.* 2nd edn. London: Butterworth. p. 253.

ELLIOTT, P. G., THURLOW, B., NEEDHAM, P. R. G. & LEWIS, M. G. (1973) The specificity of the cytoplasmic antigen in human malignant melanoma. *Eur. J. Cancer*, **9**, 607.

GORODILOVA, V. V. & HOLLINSHEAD, A. (1975) Melanoma antigens that produce cell mediated immune responses in melanoma patients. Joint U.S.–U.S.S.R. study. *Science*, **190**, 391.

GREEN, M. R., PASTEWKA, J. V. & PEACOCK, A. C. (1973) Differential staining of phospho-proteins on polyacrylamide gels with a cationic carbocyamine dye. *Anal. Biochem.*, **56**, 43.

HARTREE (1972) Determination of protein: A modification of the Lowry method that gives a linear photometric response. *Anal. Biochem.*, **48**, 422.

HILL, R. L., BEYER, T. A., REARICK, J. I., SADLER, J. E., PRIELLS, J. P. & POULSON, J. C. (1979) Glycosyl transferases in glycoconjugate biosynthesis and their use in assessing oligosaccharide structure and function. Glycoconjugates. *Proc.* 5th *Int. Symp. Kiel, Federal Republic of Germany.* Eds. Schauer *et al.* Stuttgart: Georg Thieme. p. 274.

INMAN, J. K. & DINTZIS, H. M. (1969) The derivatisation of cross linked polyacrylamide beads. Controlled introduction of functional groups for the preparation of specific-purpose, biochemical absorbents. *Biochemistry*, **8**, 4074.

IRIE, K., IRIE, R. F. & MORTON, D. L. (1975) Detection of antibody and complement complexed *in vivo* on membranes of human cancer cells by mixed hemadsorption techniques. *Cancer Res.*, **35**, 1244.

JEHN, D., NATHANSON, L., SCHWARTZ, R. S. & SKINNER, M. (1970). *In vitro* lymphocyte stimulation by a soluble tumour antigen in malignant melanoma. *N. Engl. J. Med.*, **283**, 329.

KOHN, J. (1976) Cellulose acetate electrophoresis and immunodiffusion techniques. In *Chromatographic and Electrophoretic Techniques; Vol.* 2 *Zone Electrophoresis*, 4th Edn. Ed. Smith Williams. London: Heinemann Medical Books. p. 90.

KRATBY, O., LEOPOLD, H. & STABINGER, H. (1969) Dichtemessung an Flussigkeiten und Gasen auf $10^{-6}$ g/cm$^3$ bei 0·6cm$^3$ praparatvolumen. *Z. Psychol.*, **4**, 273.

LEABACK, D. H. (1976) Concentration gradient polyacrylamide gel electrophoresis. In *Chromatographic and Electrophoretic techniques Vol.* 2 *Zone Electrophoresis* 4th Edn. Ed. Smith Williams. London: Heinemann Medical Books Ltd. p. 250.

LEWIS, M. G. (1967) Possible immunologic factors in human malignant melanoma. *Lancet*, ii, 921.

MCCABE, R. P., FARRONE, S., PELLEGRINO, M. A., KERN, D. H., HOLMES, E. C. & REISFELD, R. A. (1978) Purification and immunologic evaluation of human melanoma-associated antigens. *J. Natl Cancer Inst.*, **60**, 773.

MCBRIDE, C. M., BOWEN, J. M. & DMOCHOWSKI, L. (1972) Antinuclear antibodies in the serum of patients with malignant melanoma. *Surg. Forum*, **23**, 92.

MORTON, D. L., MALMGREN, R. A., HOLMES, E. C. & KETCHAM, A. S. (1968) Demonstration of antibodies against human malignant melanoma by immune fluorescence. *Surgery*, **64**, 233.

OUCHTERLONY, O. (1949) Antigen–antibody reactions in gels. III. Factors determining the site of the precipitate. *Arkiv. Kemi I*, **1**, 43.

PHILLIPS, B. & ROITT, I. M. (1973) Evidence for transformation of human B lymphocytes by PHA. *Nature*, **241**, 254.

RAY, P. K., THAKUR, V. S. & SUNDARAM, K. (1975) Antitumour immunity. 1. Neuraminidase-treated and X-irradiated tumour vaccine. *Eur. J. Cancer*, **11**, 1.

RIGHETTI, P. & DRYSDALE, J. W. (1971) Isoelectric focusing in polyacrylamide gels. *Biochim. Biophys. Acta*, **236**, 17.

RUOSLAHTI, E. & SAPPALA, M. (1971) Studies of carcinofetal proteins: Physical and chemical properties of human α fetoprotein. *Int. J. Cancer*, **7**, 218.

SCHEIDEGGER, J. J. & ROULEL, H. (1955) Application pratiques de la méthode immunoélectrophoretique. Premier resultats. *Praxis*, **44**, 73.

SHARIF, A., PICO, J. L., CHOQUET, C., ROSENFELD, C. & BOURILLON, R. (1978) Modifications of lectin binding on human leukemic cells after neuraminidase treatment. *Biomedicine*, **29**, 75.

STUHLMILLER, G. M., GREEN, R. W. & STEIGLER, H. G. (1978) Solubilisation and partial isolation of human melanoma tumour-associated antigens. *J. Natl Cancer Inst.*, **61**, 61.

TOMBS, M. P., SOUTER, F. & MACLAGAN, N. F. (1959) The spectrophotometric determinations of protein at 210 nm. *Biochem. J.*, **73**, 167.

VOLKERS, C., COOKE, K. B., BENNETT, C., BYROM, N., ELLIOTT, P. & WHITFIELD, P. (1978) The significance of urinary melanoma antigen excretion and the ability of thymosin to raise the level of depleted T lymphocytes *in vitro* in melanoma. *Aust. J. Surg.*, **48**, 52.

WEBER, K. & OSBOURNE, M. (1975) Protein and sodium dodecyl sulphate: Molecular weight determinations on polyacrylamide gels and related procedures. In *The Proteins*, 3rd Edn, Vol. I. Eds. Neurath & Hill. New York: Academic Press. p. 179.

YPHANTIS, D. A. (1964) Equilibrium ultracentrifugation of dilute solutions. *Biochemistry*, **3**, 297.

YON, R. J. (1972) Chromatography of lipophilic proteins on absorbents containing mixed hydrophobic and ionic groups. *Biochem. J.*, **126**, 765.

ZOLLER, M. & MATZKU, S. (1976) Antigen and antibody purification by immunoadsorption. Elimination of non-biospecifically bound proteins. *J. Immunol. Methods*, II, 287.